# Radiomic features from MRI distinguish myxomas from myxofibrosarcomas

**DOI:** 10.1186/s12880-019-0366-9

**Published:** 2019-08-15

**Authors:** Teresa Martin-Carreras, Hongming Li, Kumarasen Cooper, Yong Fan, Ronnie Sebro

**Affiliations:** 1 Department of Radiology, University of Pennsylvania, 3400 Spruce Street, Philadelphia, PA 19104 USA; 2 Department of Pathology and Laboratory Medicine, University of Pennsylvania, 3400 Spruce Street, Philadelphia, PA 19104 USA; 3 Department of Orthopedic Surgery, University of Pennsylvania, 3737 Market Street, Philadelphia, PA 19104 USA; 4 Department of Genetics, University of Pennsylvania, 421 Marie Curie Blvd, Philadelphia, PA 19104 USA; 5 Department of Epidemiology and Biostatistics, University of Pennsylvania, 421 Marie Curie Blvd, Philadelphia, PA 19104 USA

**Keywords:** Myxomas, Myxofibrosarcomas, Magnetic resonance imaging, Radiomics, Random forest

## Abstract

**Background:**

Myxoid tumors pose diagnostic challenges for radiologists and pathologists. All myxoid tumors can be differentiated from each other using fluorescent in-situ hybridization (FISH) or immunohistochemical markers, except for myxomas and myxofibrosarcomas. Myxomas and myxofibrosarcomas are rare tumors. Myxomas are benign and histologically bland, whereas myxofibrosarcomas are malignant and histologically heterogenous. Because of the histological heterogeneity, low grade myxofibrosarcomas may be mistaken for myxomas on core needle biopsies. We evaluated the performance of T1-weighted signal intensity (T1SI), tumor volume, and radiomic features extracted from magnetic resonance imaging (MRI) to differentiate myxomas from myxofibrosarcomas.

**Methods:**

The MRIs of 56 patients (29 with myxomas, 27 with myxofibrosarcomas) were analyzed. We extracted 89 radiomic features. Random forests based classifiers using the T1SI, volume features, and radiomic features were used to differentiate myxomas from myxofibrosarcomas. The classifiers were validated using a leave-one-out cross-validation. The performances of the classifiers were then compared.

**Results:**

Myxomas had lower normalized T1SI than myxofibrosaromas (*p* = 0.006) and the AUC using the T1SI was 0.713. However, the classification model using radiomic features had an AUC of 0.885 (accuracy = 0.839, sensitivity = 0.852, specificity = 0.828), and outperformed the classification models using T1SI (AUC = 0.713) and tumor volume (AUC = 0.838). The classification model using radiomic features was significantly better than the classifier using T1SI values (*p* = 0.039).

**Conclusions:**

Myxofibrosarcomas are on average higher in T1-weighted signal intensity than myxomas. Myxofibrosarcomas are larger and have shape differences compared to myxomas. Radiomic features performed best for differentiating myxomas from myxofibrosarcomas compared to T1-weighted signal intensity and tumor volume features.

**Electronic supplementary material:**

The online version of this article (10.1186/s12880-019-0366-9) contains supplementary material, which is available to authorized users.

## Background

There are several benign and malignant myxoid soft tissue tumors. Benign myxoid tumors include myxomas and angiomyxomas; and malignant myxoid tumors include fibromyxoid sarcomas, extraskeletal myxoid chondrosarcomas, ossifying fibromyxoid tumors, myxoid liposarcomas, myxoinflammatory fibroblastic tumors and myxofibrosarcomas [1]. Each myxoid neoplasms has key chromosomal translocations or immunohistochemical markers that are pathognomic for its diagnosis except for myxomas and myxofibrosarcomas [1–8]. Myxofibrosarcomas and myxomas are not associated with any particular translocation or gene expression product, and their diagnoses are based on their histological appearances [1, 8–10]. Therefore, one of the greatest diagnostic dilemmas for a pathologist lies in differentiating a myxoma from a myxofibrosarcoma, and particularly differentiating a cellular myxoma from some low-grade myxofibrosarcomas [1, 8–10]. Differentiation between these two entities is based on morphologic and histologic criteria [1, 8–10]. The challenge for pathologists to differentiate these two entities increases with core needle biopsies because of the heterogeneity of myxofibrosarcomas and because the core biopsy is subject to sampling error [11–13].

Differentiating myxomas from myxofibrosarcomas is also challenging for radiologists because these lesions have overlapping imaging features - namely they are both hyperintense on T2-weighted magnetic resonance imaging (MRI) sequences, have variable signal intensity on T1-weighted sequences, and both have heterogeneous enhancement patterns [14–18]. Myxomas have been reported to be isointense or hypointense to skeletal muscle using T1-weighted sequences [15]. Myxofibrosarcomas have been reported to be isointense to skeletal muscle on T1-weighted sequences [18, 19]. The difference is clinically significant because the surgical approach and treatment is different for these two entities. MRIs provide global assessment of the tumor whereas biopsies are limited to certain areas of the tumor; therefore, imaging is likely to be less subject to sampling error.

We hypothesized that quantitative analysis using radiomic feature extraction and classifier model analysis from preoperative MRI studies could predict whether a myxoid tumor is likely to be a myxoma or myxofibrosarcoma over volume-based and MRI signal intensity (SI) value analysis. The purpose of this study was to assess the performance of image intensity information, tumor volume, and radiomic features extracted from MRI for distinguishing myxomas from myxofibrosarcomas.

## Methods

### Patients

This single-center, retrospective case-control study was performed after institutional review board approval, with waiver of the informed consent requirement. The study was performed in compliance with the Health Insurance Portability and Accountability Act (HIPAA). A total of 56 patients who satisfied the inclusion criteria were retrospectively identified. To qualify, patients treated at our institution had to have pre-treatment MRI, and a histologically confirmed (from surgical excision and not from core needle biopsy) diagnosis of myxoma or myxofibrosarcoma between 01/01/2006 and 12/31/2017. Patients were excluded from the study if there was no pre-treatment MRI available, if the pre-treatment MRI was motion degraded or did not include a non-contrast enhanced T1-weighted MR imaging sequence, and a fluid-sensitive MR imaging sequence (STIR or T2-weighted sequence). Age and sex was recorded at the time of the histological diagnosis. The maximum size and location of the tumor was determined from evaluation of the pre-treatment MR study.

### MRI sequences

Because these tumors are extremely rare (less than 1/100,000), patients were sometimes referred to our tertiary center after initial imaging was obtained. The pre-treatment MRIs obtained on these patients were usually obtained on a variety of MRI scanners, using several different MR sequences and parameters. We focused on T1 sequences because this was the most available sequence (all tumor imaging MRI protocols had T1 sequences without fat saturation).

Pre-treatment MRIs were performed using either 0.6 T (Fonar Corp), 1.2 T (Hitachi Oasis), 1.5 T (Siemens Magnetom Espree; Siemens Avanto; General Electric Medical Systems Optima; General Electric Medical Systems Signa Excite) or 3 T (Siemens Verio; Siemens Symphony) systems. T1-weighted sequences were as follows: 0.6 T (repetition time (TR) 414 ms, echo time (TE) 20 ms, slice thickness 5 mm, interslice gap 0 mm, acquisition matrix 1024 × 200); 1.2 T (TR 545, TE 12, slice thickness 4 mm, interslice gap 1 mm, acquisition matrix 256 × 192); 1.5 T (TR 400–600 ms, TE 10–20 ms, slice thickness 3–5 mm, interslice gap 0.5–1 mm) and 3 T (TR 560–700 ms, TE 9–23 ms, slice thickness 4–6 mm, interslice gap 1–1.5 mm, and acquisition matrix 256–320 × 204–224).

### Tissue segmentation

ITK-SNAP software (open source, http://www.itksnap.org/) was used for manual segmentation of MR images. In all cases, the tumor was segmented on the unenhanced T1-weighted sequences. A reference region-of-interest (ROI) was drawn in the adjacent normal muscle on unenhanced T1-weighted MR images for image intensity normalization purposes. Adjacent normal muscle had to have no signal abnormality on fluid-sensitive sequences (T2-weighted with fat saturation (T2w FS)/short tau inversion recovery (STIR)) because tumor cells have been found in the peritumoral edema and these tumor cells may affect the T1 signal intensity measured [18]. Reference ROIs in the muscle were drawn to avoid tendons. This reference ROI had to be greater than 35 mm^2^. Segmentations were done by radiology resident and verified/corrected by a senior musculoskeletal radiologist.

### Image intensity normalization

To adjust for differences in MRI protocols and field strengths the T1-weighted SI was normalized. The normalized intensity map was calculated as In = I/Iref× 255, where I was the original T1 intensity, and Iref was the mean intensity value within the reference ROI. The normalized intensity map was resampled at a spatial resolution of 1 × 1 × 1 mm^3^ before radiomic feature extraction.

### Radiomic feature extraction

**Fig. 1:**
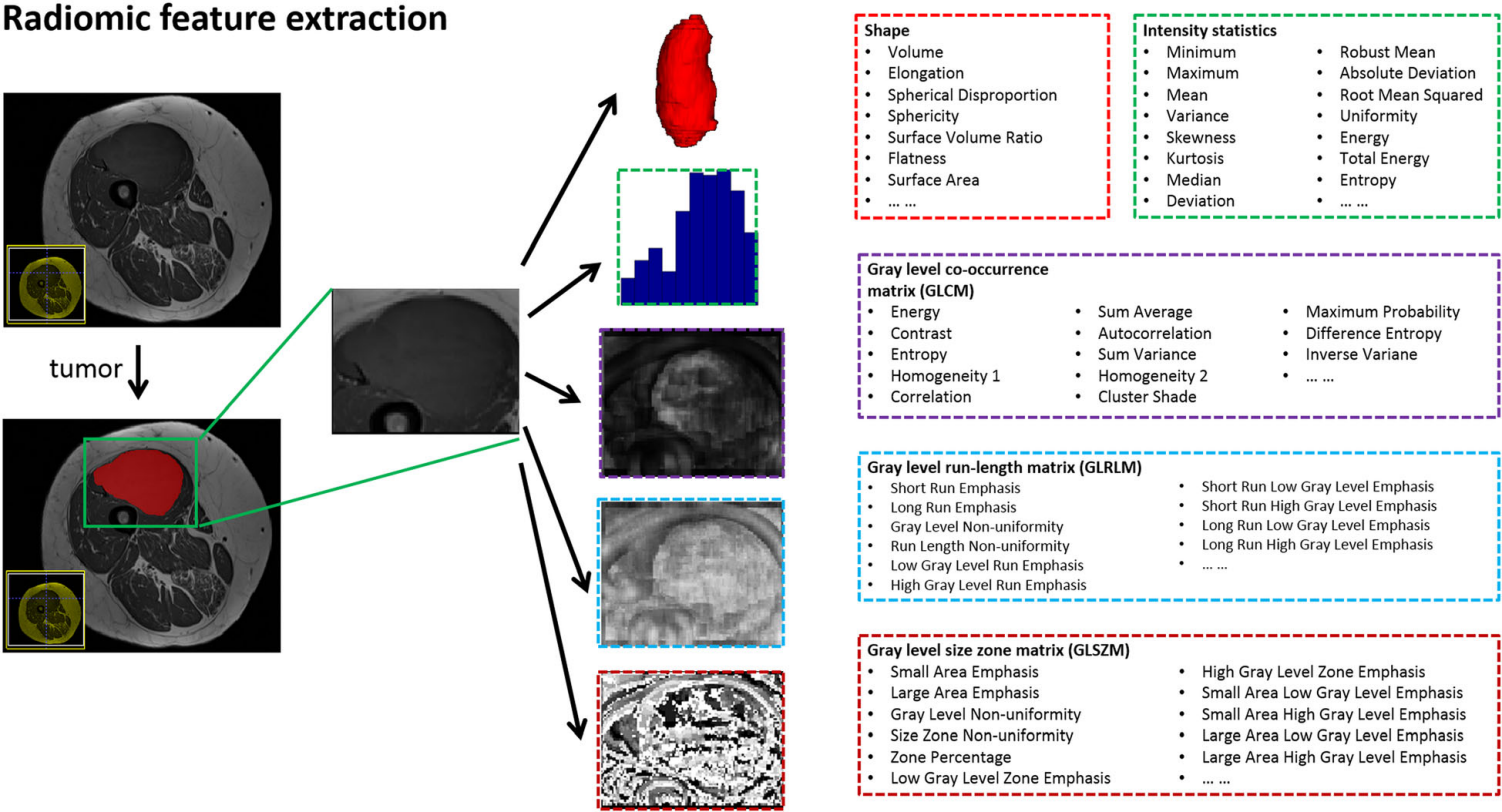
Extraction of radiomic features from T1 MRI

Radiomic features of the tumors were extracted from the normalized intensity map of the T1 sequence for each patient. In particular, 10 morphologic/volumetric features (volume features) were extracted from each tumor region. Moreover, 79 texture features of the tumor region were extracted from the normalized intensity map, including the first order features, gray level co-occurrence matrix (GLCM) features, gray level size zone matrix (GLSZM) features, and gray level run length matrix (GLRLM) features. All of the features used and their definitions are provided in the Additional file 1: Table S1. In total, 89 radiomic features of the tumor were extracted for each subject. We adopted the fixed bin size strategy for the grey-level discretization with the bin size set to 5. The feature extraction was carried out in the 3D space and 26-connected neighborhood was adopted. The flowchart for radiomic feature extraction is illustrated in Fig. 1.

### Classification

A random forests based classifier was built upon the radiomic features for distinguishing myxoma from myxofibrosarcomas. The classifiers built upon radiomic features were compared with those based on intensity and volume features in terms of their performance. The number of trees and the minimum leaf size of the random forests classifiers were set to 500 and 3, respectively. The classifiers were validated using a leave-one-out cross-validation. Classification accuracy, sensitivity, specificity, and area under the receiver operating characteristic curve (AUC) were used to evaluate the classification performance. Moreover, the importance for each radiomic feature regarding the prediction was estimated using the out-of-bag permuted predictor delta error. We adopted the implementation for random forests (TreeBagger) in Matlab (R2013a) to train the classifier.

### Histopathology

Histological analysis on the excisional sample was performed by a pathologist. There were 29 patients with myxomas, 5 (18.5%) patients with grade 1 myxofibrosarcomas, 6 (22.2%) patients with grade 2 myxofibrosarcomas and 16 (59.3%) patients with grade 3 myxofibrosarcomas.

### Statistics

Statistical computing was performed using R (version 3.4.0) [20]. Variables were compared using Wilcoxon-Rank sum tests for quantitative variables and chi-squared tests for qualitative variables. Receiver operating characteristics (ROC) curves and area under the curve (AUC) analyses were obtained using the plotROC package. DeLong test (included in the Daim package) was used to compare AUCs of the classifiers built upon different sets of features. All tests were two-sided, and a *P* value less than 0.05 was considered statistically significant.

## Results

There were a total of 56 patients identified: 29 with myxomas, 27 with myxofibrosarcomas. Subject demographic and clinical variables are shown in Table 1. There was a higher proportion of female patients with myxomas than myxofibrosarcomas, but the difference was not significantly different (*p* = 0.186 by Pearson’s Chi-squared test). None of the patients had a diagnosis of fibrous dysplasia.

**Table 1:** Study demographics

	Myxomas (*N* = 29)	Myxofibrosarcomas (*N* = 27)	*P*-value
Age in years (SD)	57.0 (12.1)	60.7 (15.6)	0.330
No. of women (%)	22 (75.9%)	15 (55.6%)	0.186
Maximum size (cm)	3.61 (1.3)	10.01 (8.0)	< 0.001
Location			0.012*
Shoulder	6	2	
Arm	0	2	
Forearm	0	3	
Chin	1	0	
Buttock	5	0	
Thigh	10	12	
Leg	1	4	
Foot	2	0	
Pelvis	3	0	
Elbow	1	1	
Knee	0	3	

Clinicodemographic characteristics of patients with myxomas and myxofibrosarcomas

*P-value based on Fisher’s exact test

**Fig. 2:**
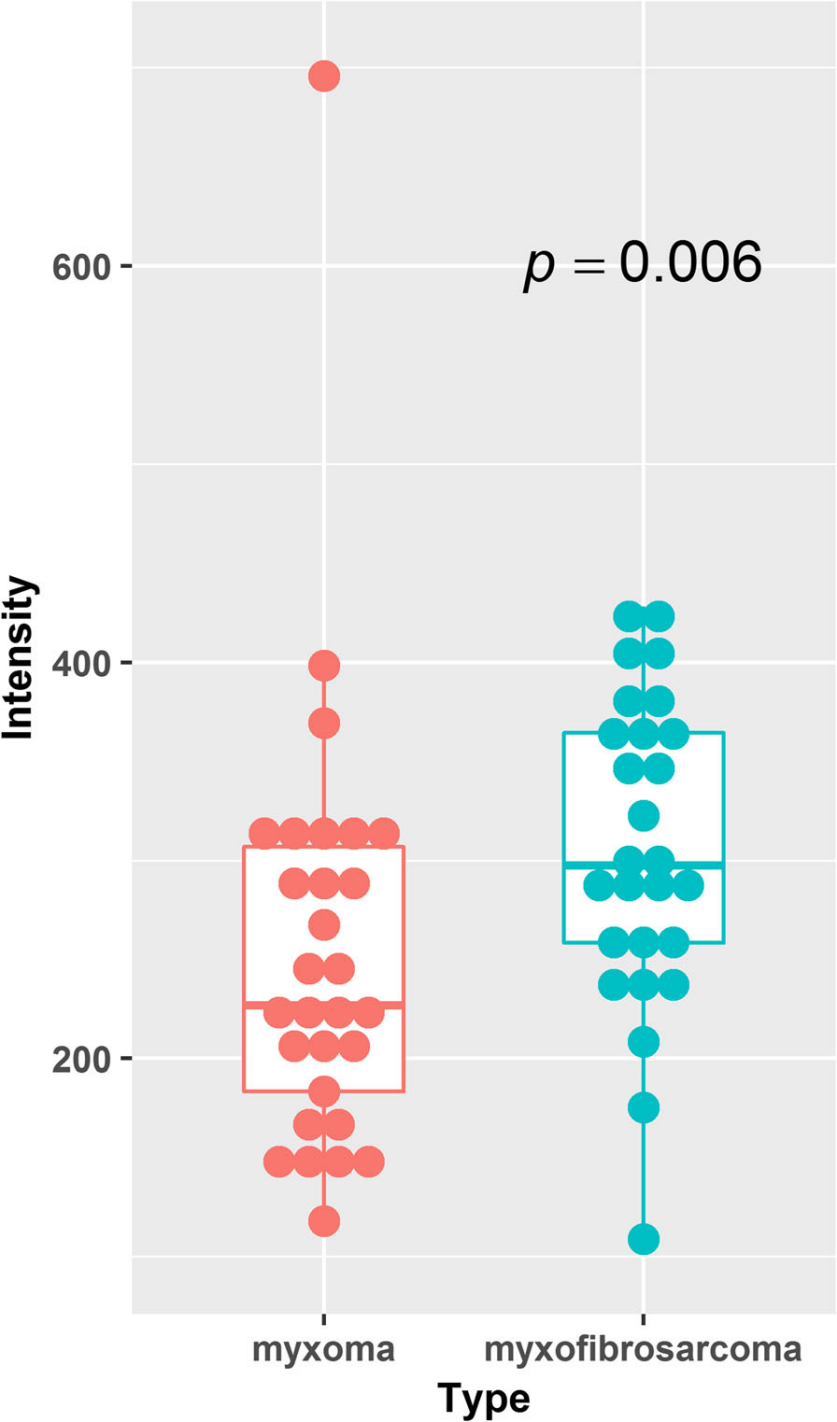
Boxplots of normalized T1-weighted signal intensity for myxoma (red) and myxofibrosarcoma (green) tumors

**Fig. 3:**
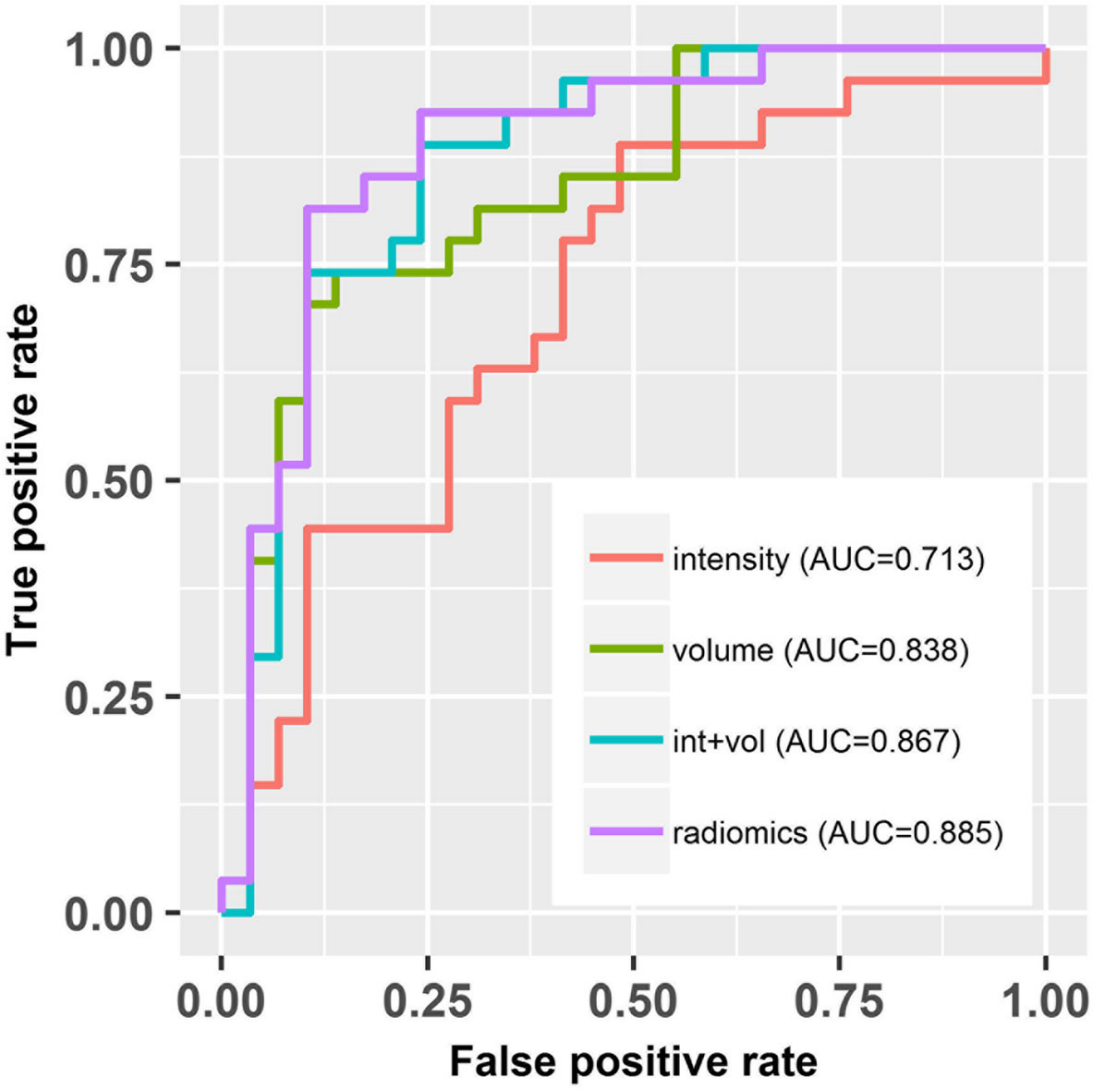
Receiver-operative characteristic (ROC) curves of classifiers built upon image intensity, volume features, imaging intensity + volume features, and radiomic features

**Fig. 4:**
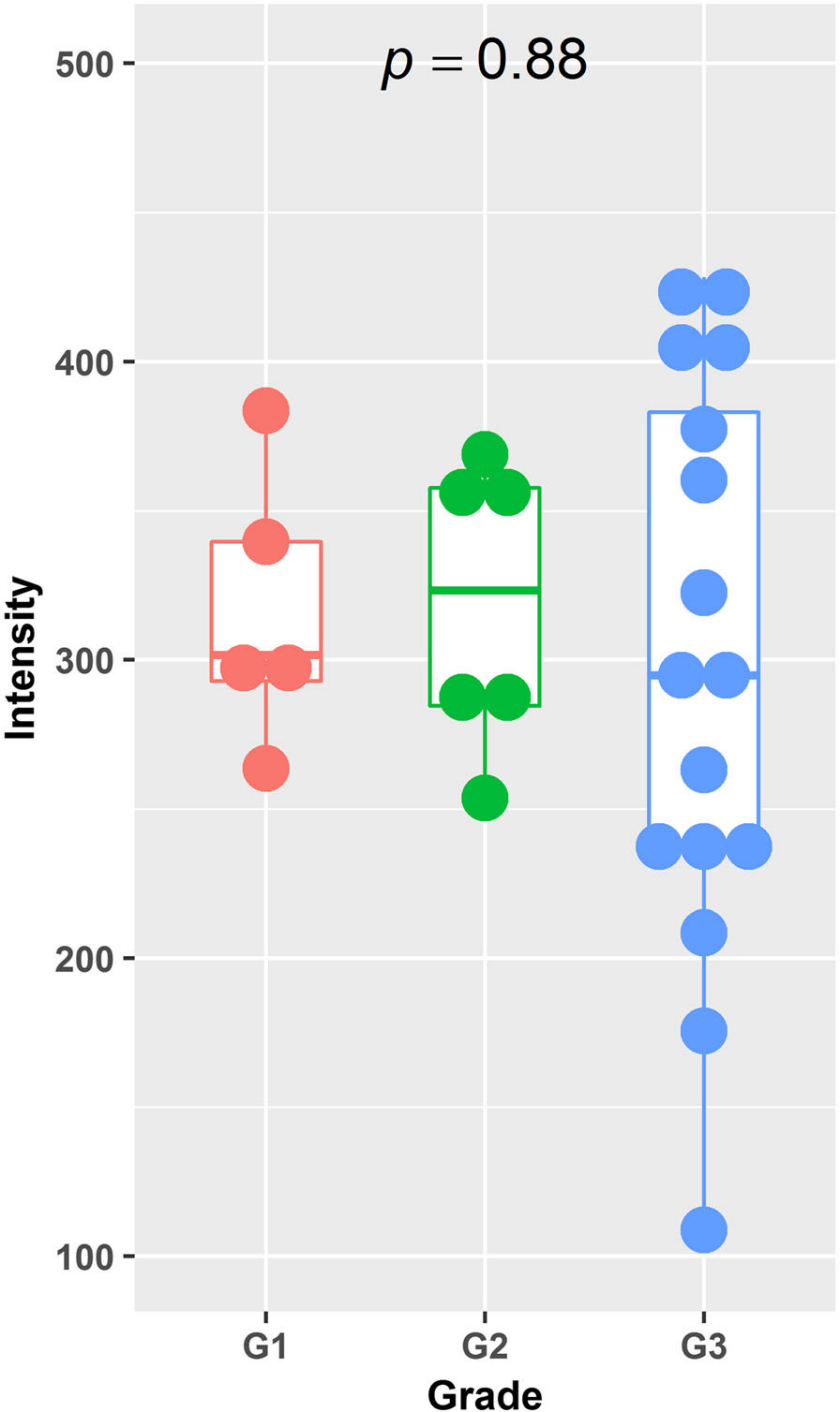
Boxplots of normalized T1-weighted signal intensity for grade1 (red), grade 2 (green) and grade 3 (blue) myxofibrosarcomas

**Fig. 5:**
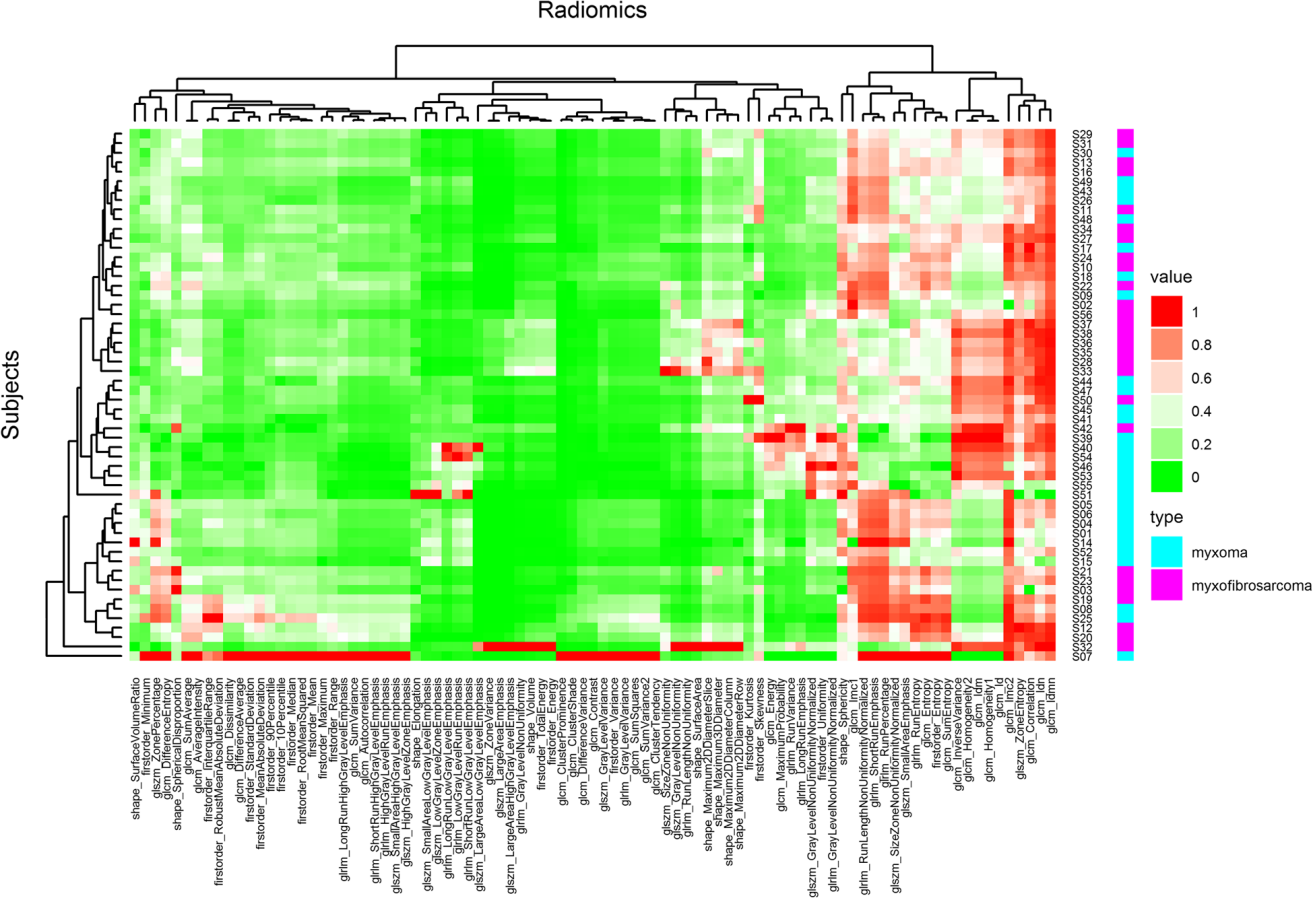
Radiomics heat map. The *x* axis refers to radiomic features, and *y* axis refers to different subjects. Dendrograms regarding radiomics and subjects were displayed to facilitate the visualization of the radiomic patterns. The type of tumor for each subject was indicated by different colors (magenta/cyan)

As shown in Fig. 2, myxomas had lower normalized T1-weighted signal intensity values than myxofibrosaromas (*p* = 0.006, Wilcoxon rank sum test), and AUC was 0.713 as shown in Fig. 3. Figure 4 shows the T1 SI of the myxofibrosarcomas by tumor grade. There was no substantial difference in the T1 SI between the myxofibrosarcomas by tumor grade (Kruskal-Wallis *p* = 0.88). The radiomics features for all the subjects were demonstrated in Fig. 5. The classification model built upon radiomic features obtained an AUC of 0.885 (accuracy = 0.839, sensitivity = 0.852, specificity = 0.828), which outperformed the classification model built upon the T1SI values (*p* = 0.039, DeLong test), and the classification model built upon volume features (AUC = 0.838, *p* = 0.285 by DeLong test) as shown in Fig. 3.

**Fig. 6:**
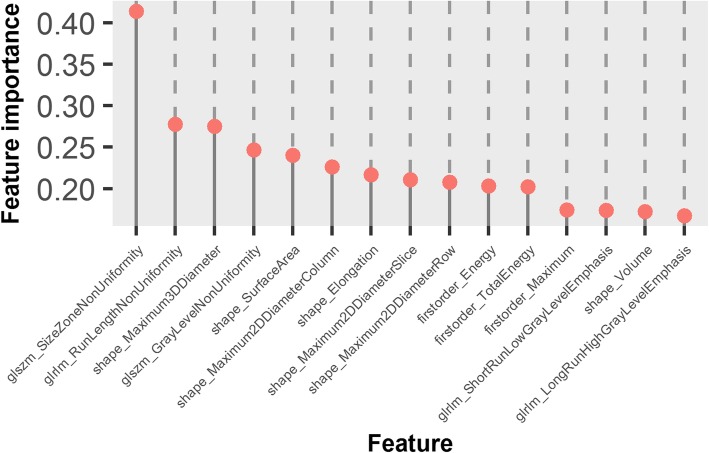
Top 15 radiomic features with high importance in the random forests based classifier

To investigate how different features contributed the classification, the top 15 features with high importance regarding classification are demonstrated in Fig. 6. Seven of them were shape-based measures, indicating high association between tumor type and their morphologic properties. This also supported the result that the volume features based classifier had better performance than that based on intensity. The remaining 8 were texture features, and the feature GLSZM_SizeZoneNonUniformity had the highest importance, indicating that texture features were more discriminative, and could provide complementary information to the shape-based features. This suggests that myxofibrosarcomas were more heterogeneous on T1-weighted sequences than myxomas.

## Discussion

The results show that the T1SI of myxofibrosarcomas are on average higher than that of myxomas, however, there was significant T1SI overlap for both lesions. We hypothesize that a more cellular tumor has higher protein content, and would therefore result in increased T1 shortening (higher T1 signal) as our results have shown. An alternative explanation is that these malignant lesions may contain small foci of hemorrhage. Myxofibrosarcomas also tended to have volumetric features that were slightly different than myxomas. Myxofibrosarcomas have been noted to have a “tail sign” and have a known propensity for spreading along the myofascial planes [18, 21]. This feature may have been detected as part of the volumetric features.

Radiomic (texture) features were the best for differentiating myxofibrosarcomas from myxomas. Quantitative analysis using a classification model based on radiomic features outperformed the classification models using volume-based and T1SI value analysis. Myxomas tend to be more paucicellular and bland (unless a cellular myxoma), and therefore have less T1 signal heterogeneity. T1-weighted signal heterogeneity of myxofibrosarcomas was greater than that for myxomas, and we speculate that the T1-weighted signal intensity heterogeneity mirrors the intrinsic histologic tumor heterogeneity seen in myxofibrosarcomas and possibly intratumoral hemorrhage.

Prior reports support our findings. Myxomas have been shown to be hypointense to skeletal muscle on T1-weighted sequences [15, 16], whereas myxofibrosarcomas have been shown to be more isointense to skeletal muscle on T1-weighted sequences [19]. However, no reports have shown that volumetric and radiomic texture features can be utilized to differentiate myxomas from myxofibrosarcomas from preoperative MRIs.

The results have potential significant clinical implications. Core biopsies are limited by the fact that these lesions are heterogeneous, and the sample cannot entirely represent the lesion’s functional and histologic properties [22]. Image-guided core biopsies targeting areas of necrosis in one of the samples may even add to the correct grade specifically in myxofibrosarcoma [23]. Radiomics offers a non-invasive, cost-effective method for assessment of a lesion’s entire tumor spatial and temporal heterogeneity [22].

We have shown that MRI image-derived radiomic features can quite accurately differentiate two extremely rare tumor types (myxomas and myxofibrosarcomas) which are challenging for pathologists and radiologists. This is particularly exciting because these tumors are so rare, most radiologists rarely encounter these tumors in daily practice, so most radiologists have limited experience in differentiating these two entities.

Radiomic feature extraction and analysis has been applied broadly to other subspecialties in radiology with successful applications in discerning molecular alterations in tumors, predicting and stratifying tumor response to therapy, and to determine patient prognosis [24–29]. However, literature on musculoskeletal applications of radiomics is scarce. To our knowledge, this is the first study to use radiomic feature extraction and classifier prediction models for this purpose.

The study had some limitations. First, it was retrospective in nature, and subject to ascertainment bias. Myxomas and myxofibrosarcomas are rare tumors (1–4 cases per million people) and this analysis represents one of the largest analyses in the published literature of myxomas and myxofibrosarcomas. Another limitation is that pre-treatment MRIs were obtained using variable parameters and field strengths; however, this was adjusted for by using image intensity normalization. We found that all tumor MRI sequences typically included a T1-weighted sequence, which was why the analysis was restricted to T1-weighted sequences. This makes our findings more broadly applicable to clinical practices which use T1-weighted sequences in their tumor protocols. It is conceivable that there would be additional discriminative information contained in T2 or STIR and post-contrast sequences. We did not analyze the T2/STIR sequences because these were not always available (some patients had T2-weighted sequences, others had proton density-weighted sequences and others had STIR sequences), which would have left us with a much smaller sample size with limited power due to missing data. Myxofibrosarcomas often have perilesional edema and often have a tail-sign on T2-weighted sequences, which would likely add to the discriminatory ability of the model. Additional research is required to assess whether additional information can be obtained from the T2/STIR sequences to differentiate myxomas from myxofibrosarcomas. We did not analyze the contrast-enhanced T1-weighted sequences because not all patients had contrast enhanced studies and because the time from injection of contrast to imaging was not uniform across patients and we thought this would introduce more noise into the analysis and not be definitive.

## Conclusion

In summary, we have demonstrated that radiomic features from T1-weighted sequences can provide better discriminative information in distinguishing myxoma from myxofibrosarcomas compared to T1-weighted signal intensity values and tumor volume.

## Additional file

Additional file 1: Radiomic features adopted in this study. This file shows how all of the radiomic features used in this study were calculated. (DOCX 28 kb)

## Data Availability

The datasets used and/or analyzed during the current study are available from the corresponding author on reasonable request. *Image Attribution*: All images are our own.

## Abbreviations

AUC: Area under the curve
GLCM: Gray level co-occurrence matrix
GLRLM: Gray level run length matrix
GLSZM: Gray level size zone matrix
GLSZM_SizeZoneNonUniformity: Gray level size zone matrix size zone non-uniformity
HIPAA: Health Insurance Portability and Accountability Act
MR: Magnetic resonance
MRI: Magnetic resonance imaging
ROC: Receiver operator characteristic
STIR: Short tau inversion recovery
T1SI: T1-weighted signal intensity
