# Supplementary material for: Radiomic features from MRI distinguish myxomas from myxofibrosarcomas
Source: BMC Med Imaging. 2019 Aug 15;19:67. doi: 10.1186/s12880-019-0366-9 (PMC6694512; doi:10.1186/s12880-019-0366-9)
Supplement: Supplementary file 1 — Radiomic features adopted in this study. This file shows how all of the radiomic features used in this study were calculated. (DOCX 28 kb) [file 12880_2019_366_MOESM1_ESM.docx]

**Supplementary data**

**Radiomic features adopted in this study**

All the radiomic features used in this study are listed in table S1, and more details could be found at http://pyradiomics.readthedocs.io/en/1.1.1/features.html.

Table S1. Radiomic features adopted in this study.

| Morphometry | Volume ($V$)  Elongation $\frac{\lambda_{longest}}{\lambda_{intermediate}}$  Spherical Disproportion $\frac{A}{\sqrt[3]{36\pi V^{2}}}$  Sphericity $\frac{\sqrt[3]{36\pi V^{2}}}{A}$  Surface Volume Ratio $\frac{A}{V}$  Surface Area ($A$)  Maximum 3D Diameter  Maximum 2D Diameter axial  Maximum 2D Diameter coronal  Maximum 2D Diameter sagittal  where $\lambda_{longest}$, $\lambda_{intermediate}$, and $\lambda_{shortest}$ are the lengths of the largest, second largest and smallest principal component axes. |
| --- | --- |
| First-order statistics | Minimum $min(X)$  Maximum $max(X)$  Mean $\bar{X}=\frac{1}{N}\sum_{i=1}^{N} X(i)$  Standard Deviation $\sqrt{\frac{1}{N}\sum_{i=1}^{N} {(X\left( i \right)-\bar{X})}^{2}}$  Variance $\frac{1}{N}\sum_{i=1}^{N} {(X\left( i \right)-\bar{X})}^{2}$  Skewness $\frac{\frac{1}{N}\sum_{i=1}^{N} {(X\left( i \right)-\bar{X})}^{3}}{{(\sqrt{\frac{1}{N}\sum_{i=1}^{N} {(X\left( i \right)-\bar{X})}^{2}})}^{3}}$  Kurtosis $\frac{\frac{1}{N}\sum_{i=1}^{N} {(X\left( i \right)-\bar{X})}^{4}}{{(\frac{1}{N}\sum_{i=1}^{N} {(X\left( i \right)-\bar{X})}^{2})}^{2}}$  Median  Range $max(X)-min(X)$  10-th Percentile $P_{10}$  90-th Percentile $P_{90}$  Interquartile Range $P_{75}-P_{25}$  Mean Absolute Deviation $\frac{1}{N}\sum_{i=1}^{N} \left\vert X\left( i \right)-\bar{X} \right\vert$  Robust Mean Absolute Deviation $\frac{1}{N_{10-90}}\sum_{i=1}^{N_{10-90}} \left\vert X_{10-90}\left( i \right)-\bar{X}_{10-90} \right\vert$  Root Mean Squared $\sqrt{\frac{1}{N}\sum_{i=1}^{N} {X\left( i \right)}^{2}}$  Uniformity $\sum_{i=1}^{N_{l}} {p\left( i \right)}^{2}$  Energy $\sum_{i=1}^{N} {X\left( i \right)}^{2}$  Total Energy $V_{voxel}\sum_{i=1}^{N} {X\left( i \right)}^{2}$  Entropy $-\sum_{i=1}^{N_{l}} p(i)\log_{2} (p\left( i \right)+\epsilon)$  where $X$ denotes the 3D image matrix with $N$ voxels, $p(i)$ is the normalized first order histogram with $N_{l}$ intensity levels, $V_{voxel}$ is the volume of the voxel in ${mm}^{3}$, $P_{d}$is the $d$-th percentile of the image matrix, $X_{10-90}$ denotes the subset of image matrix with intensity levels in between the 10-th and 90-th percentile, and $\epsilon$ is an arbitrary small positive number. |
| Gray level co-occurrence matrix (GLCM) | Energy $\sum_{i=1}^{N_{g}} \sum_{j=1}^{N_{g}} {p(i,j)}^{2}$  Contrast $\sum_{i=1}^{N_{g}} \sum_{j=1}^{N_{g}} {(i-j)}^{2}p(i,j)$  Entropy $-\sum_{i=1}^{N_{g}} \sum_{j=1}^{N_{g}} p(i,j)\log_{2} (p\left( i,j \right)+\epsilon)$  Homogeneity 1 $\sum_{i=1}^{N_{g}} \sum_{j=1}^{N_{g}} \frac{p(i,j)}{1+\left\vert i-j \right\vert}$  Correlation $\frac{\sum_{i=1}^{N_{g}} \sum_{j=1}^{N_{g}} p(i,j)ij-u_{x}u_{y}}{\sigma_{x}\sigma_{y}}$  Sum Average (SA) $\sum_{k=2}^{{2N}_{g}} kp_{x+y}(k)$  Autocorrelation $\sum_{i=1}^{N_{g}} \sum_{j=1}^{N_{g}} p(i,j)ij$  Sum Variance $\sum_{k=2}^{{2N}_{g}} {(k-SE)}^{2}p_{x+y}(k)$  Homogeneity 2 $\sum_{i=1}^{N_{g}} \sum_{j=1}^{N_{g}} \frac{p(i,j)}{1+\left\vert i-j \right\vert^{2}}$  Cluster Shade $\sum_{i=1}^{N_{g}} \sum_{j=1}^{N_{g}} {(i+j-u_{x}-u_{y})}^{3}p(i,j)$  Maximum Probability $max(p(i,j))$  Inverse Difference Moment Normalized $\sum_{i=1}^{N_{g}} \sum_{j=1}^{N_{g}} \frac{p(i,j)}{1+\frac{\left\vert i-j \right\vert^{2}}{N_{g}^{2}}}$  Difference Entropy $\sum_{k=0}^{N_{g}-1} p_{x-y}(k)\log_{2} (p_{x-y}\left( k \right)+\epsilon)$  Inverse Variance $\sum_{i=1}^{N_{g}} \sum_{j=1}^{N_{g}} \frac{p(i,j)}{\left\vert i-j \right\vert^{2}}, i\neq j$  Dissimilarity $\sum_{i=1}^{N_{g}} \sum_{j=1}^{N_{g}} p(i,j)\left\vert i-j \right\vert$  Difference Variance $\sum_{k=0}^{N_{g}-1} {(1-DA)}^{2}p_{x-y}(k)$  Inverse Difference Normalized $\sum_{i=1}^{N_{g}} \sum_{j=1}^{N_{g}} \frac{p(i,j)}{1+\frac{\left\vert i-j \right\vert}{N_{g}}}$  Inverse Difference moment $\sum_{i=1}^{N_{g}} \sum_{j=1}^{N_{g}} \frac{p(i,j)}{1+\left\vert i-j \right\vert^{2}}$  Sum Entropy (SE) $\sum_{k=2}^{{2N}_{g}} p_{x+y}(k)\log_{2} (p_{x+y}\left( k \right)+\epsilon)$  Sum of Squares $\sum_{i=1}^{N_{g}} \sum_{j=1}^{N_{g}} {(i-u_{x})}^{2}p(i,j)$  Cluster Prominence $\sum_{i=1}^{N_{g}} \sum_{j=1}^{N_{g}} {(i+j-u_{x}-u_{y})}^{4}p(i,j)$  Informal Measure of Correlation 1 $\frac{HXY-HXY1}{max\{HX,HY\}}$  Informal Measure of Correlation 2 $\sqrt{1-e^{-2(HXY2-HXY)}}$  Difference Average (DA) $\sum_{k=0}^{N_{g}-1} kp_{x-y}(k)$  Inverse Difference $\sum_{i=1}^{N_{g}} \sum_{j=1}^{N_{g}} \frac{p(i,j)}{1+\left\vert i-j \right\vert}$  Cluster Tendency $\sum_{i=1}^{N_{g}} \sum_{j=1}^{N_{g}} {(i+j-u_{x}-u_{y})}^{2}p(i,j)$  Sum Variance 2 $\sum_{k=2}^{{2N}_{g}} {(k-SA)}^{2}p_{x+y}(k)$  Average Intensity $\sum_{i=1}^{N_{g}} \sum_{j=1}^{N_{g}} p(i,j)i$  where $p\left( i,j \right)$ is the normalized co-occurrence matrix, $N_{g}$is the number of intensity levels, $p_{x}\left( i \right)=\sum_{j=1}^{N_{g}} p(i,j)$, $p_{y}\left( j \right)=\sum_{i=1}^{N_{g}} p(i,j)$, $u_{x}=\sum_{i=1}^{N_{g}} \sum_{j=1}^{N_{g}} p(i,j)i$, $u_{y}=\sum_{i=1}^{N_{g}} \sum_{j=1}^{N_{g}} p(i,j)j$, $\sigma_{x}$and $\sigma_{y}$are the standard deviation of $p_{x}$ and $p_{y}$, $p_{x+y}\left( k \right)=\sum_{i=1}^{N_{g}} \sum_{j=1}^{N_{g}} p\left( i,j \right)$ where $i+j=k$, $p_{x-y}\left( k \right)=\sum_{i=1}^{N_{g}} \sum_{j=1}^{N_{g}} p\left( i,j \right)$ where $\left\vert i-j \right\vert=k$, $HX=-\sum_{i=1}^{N_{g}} p_{x}(i)\log_{2} (p_{x}\left( i \right)+\epsilon)$, $HY=-\sum_{j=1}^{N_{g}} p_{y}(j)\log_{2} (p_{y}\left( j \right)+\epsilon)$, $HXY=-\sum_{i=1}^{N_{g}} \sum_{j=1}^{N_{g}} p(i,j)\log_{2} (p\left( i,j \right)+\epsilon)$, $HXY1=-\sum_{i=1}^{N_{g}} \sum_{j=1}^{N_{g}} p(i,j)\log_{2} (p_{x}(i)p_{y}(j)+\epsilon)$, $HXY2=-\sum_{i=1}^{N_{g}} \sum_{j=1}^{N_{g}} p_{x}(i)p_{y}(j)\log_{2} (p_{x}(i)p_{y}(j)+\epsilon)$, and $\epsilon$ is an arbitrary small positive number. |
| Gray level run length matrix (GLRLM) | Short Run Emphasis $\frac{\sum_{i=1}^{N_{g}} \sum_{j=1}^{N_{r}} \frac{P(i,j\vert\theta)}{j^{2}}}{\sum_{i=1}^{N_{g}} \sum_{j=1}^{N_{r}} P(i,j\vert\theta)}$  Long Run Emphasis $\frac{\sum_{i=1}^{N_{g}} \sum_{j=1}^{N_{r}} P(i,j\vert\theta)j^{2}}{\sum_{i=1}^{N_{g}} \sum_{j=1}^{N_{r}} P(i,j\vert\theta)}$  Gray Level Non-uniformity $\frac{\sum_{i=1}^{N_{g}} {(\sum_{j=1}^{N_{r}} P(i,j\vert\theta))}^{2}}{\sum_{i=1}^{N_{g}} \sum_{j=1}^{N_{r}} P(i,j\vert\theta)}$  Run Length Non-uniformity $\frac{\sum_{j=1}^{N_{r}} {(\sum_{i=1}^{N_{g}} P(i,j\vert\theta))}^{2}}{\sum_{i=1}^{N_{g}} \sum_{j=1}^{N_{r}} P(i,j\vert\theta)}$  Low Gray Level Run Emphasis $\frac{\sum_{i=1}^{N_{g}} \sum_{j=1}^{N_{r}} \frac{P(i,j\vert\theta)}{i^{2}}}{\sum_{i=1}^{N_{g}} \sum_{j=1}^{N_{r}} P(i,j\vert\theta)}$  High Gray Level Run Emphasis $\frac{\sum_{i=1}^{N_{g}} \sum_{j=1}^{N_{r}} P(i,j\vert\theta)i^{2}}{\sum_{i=1}^{N_{g}} \sum_{j=1}^{N_{r}} P(i,j\vert\theta)}$  Short Run Low Gray Level Emphasis $\frac{\sum_{i=1}^{N_{g}} \sum_{j=1}^{N_{r}} \frac{P(i,j\vert\theta)}{{i^{2}j}^{2}}}{\sum_{i=1}^{N_{g}} \sum_{j=1}^{N_{r}} P(i,j\vert\theta)}$  Short Run High Gray Level Emphasis $\frac{\sum_{i=1}^{N_{g}} \sum_{j=1}^{N_{r}} \frac{P(i,j\vert\theta)i^{2}}{j^{2}}}{\sum_{i=1}^{N_{g}} \sum_{j=1}^{N_{r}} P(i,j\vert\theta)}$  Long Run Low Gray Level Emphasis $\frac{\sum_{i=1}^{N_{g}} \sum_{j=1}^{N_{r}} \frac{P(i,j\vert\theta)j^{2}}{i^{2}}}{\sum_{i=1}^{N_{g}} \sum_{j=1}^{N_{r}} P(i,j\vert\theta)}$  Long Run High Gray Level Emphasis $\frac{\sum_{i=1}^{N_{g}} \sum_{j=1}^{N_{r}} P(i,j\vert\theta)i^{2}j^{2}}{\sum_{i=1}^{N_{g}} \sum_{j=1}^{N_{r}} P(i,j\vert\theta)}$  Gray Level Variance $\sum_{i=1}^{N_{g}} \sum_{j=1}^{N_{r}} P(i,j\vert\theta){(i-u)}^{2}$ where $u=\sum_{i=1}^{N_{g}} \sum_{j=1}^{N_{r}} P(i,j\vert\theta)i$  Run Variance $\sum_{i=1}^{N_{g}} \sum_{j=1}^{N_{r}} P(i,j\vert\theta){(j-u)}^{2}$ where $u=\sum_{i=1}^{N_{g}} \sum_{j=1}^{N_{r}} P(i,j\vert\theta)j$  Run Entropy $-\sum_{i=1}^{N_{g}} \sum_{j=1}^{N_{r}} P(i,j\vert\theta)\log_{2} (P\left( i,j \vert\theta\right)+\epsilon)$  Run Percentage $\sum_{i=1}^{N_{g}} \sum_{j=1}^{N_{r}} \frac{P(i,j\vert\theta)}{N_{p}}$  Gray Level Non-uniformity Normalized $\frac{\sum_{i=1}^{N_{g}} {(\sum_{j=1}^{N_{r}} P(i,j\vert\theta))}^{2}}{\sum_{i=1}^{N_{g}} \sum_{j=1}^{N_{r}} {P(i,j\vert\theta)}^{2}}$  Run Length Non-uniformity Normalized $\frac{\sum_{j=1}^{N_{r}} {(\sum_{i=1}^{N_{g}} P(i,j\vert\theta))}^{2}}{\sum_{i=1}^{N_{g}} \sum_{j=1}^{N_{r}} {P(i,j\vert\theta)}^{2}}$  where $P(i,j\vert\theta)$ is the run length matrix for direction $\theta$, $N_{g}$ is the number of intensity levels, $N_{r}$ is the number of run lengths, $N_{p}$ is the number of voxels, and $\epsilon$ is an arbitrary small positive number. |
| Gray level size zone matrix (GLSZM) | Small Area Emphasis $\frac{\sum_{i=1}^{N_{g}} \sum_{j=1}^{N_{s}} \frac{P(i,j)}{j^{2}}}{\sum_{i=1}^{N_{g}} \sum_{j=1}^{N_{s}} P(i,j)}$  Large Area Emphasis $\frac{\sum_{i=1}^{N_{g}} \sum_{j=1}^{N_{s}} P(i,j)j^{2}}{\sum_{i=1}^{N_{g}} \sum_{j=1}^{N_{s}} P(i,j)}$  Gray Level Non-uniformity $\frac{\sum_{i=1}^{N_{g}} {(\sum_{j=1}^{N_{s}} P(i,j))}^{2}}{\sum_{i=1}^{N_{g}} \sum_{j=1}^{N_{s}} P(i,j)}$  Size Zone Non-uniformity $\frac{\sum_{j=1}^{N_{s}} {(\sum_{i=1}^{N_{g}} P(i,j))}^{2}}{\sum_{i=1}^{N_{g}} \sum_{j=1}^{N_{s}} P(i,j)}$  Zone Percentage $\sum_{i=1}^{N_{g}} \sum_{j=1}^{N_{s}} \frac{P(i,j)}{N_{p}}$  Low Gray Level Zone Emphasis $\frac{\sum_{i=1}^{N_{g}} \sum_{j=1}^{N_{s}} \frac{P(i,j)}{i^{2}}}{\sum_{i=1}^{N_{g}} \sum_{j=1}^{N_{s}} P(i,j)}$  High Gray Level Zone Emphasis $\frac{\sum_{i=1}^{N_{g}} \sum_{j=1}^{N_{s}} P(i,j)i^{2}}{\sum_{i=1}^{N_{g}} \sum_{j=1}^{N_{s}} P(i,j)}$  Small Area Low Gray Level Emphasis $\frac{\sum_{i=1}^{N_{g}} \sum_{j=1}^{N_{s}} \frac{P(i,j)}{{i^{2}j}^{2}}}{\sum_{i=1}^{N_{g}} \sum_{j=1}^{N_{s}} P(i,j)}$  Small Area High Gray Level Emphasis $\frac{\sum_{i=1}^{N_{g}} \sum_{j=1}^{N_{s}} \frac{P(i,j)i^{2}}{j^{2}}}{\sum_{i=1}^{N_{g}} \sum_{j=1}^{N_{s}} P(i,j)}$  Large Area Low Gray Level Emphasis $\frac{\sum_{i=1}^{N_{g}} \sum_{j=1}^{N_{s}} \frac{P(i,j)j^{2}}{i^{2}}}{\sum_{i=1}^{N_{g}} \sum_{j=1}^{N_{s}} P(i,j)}$  Large Area High Gray Level Emphasis $\frac{\sum_{i=1}^{N_{g}} \sum_{j=1}^{N_{s}} P(i,j)i^{2}j^{2}}{\sum_{i=1}^{N_{g}} \sum_{j=1}^{N_{s}} P(i,j)}$  Gray Level Variance $\sum_{i=1}^{N_{g}} \sum_{j=1}^{N_{s}} P(i,j){(i-u)}^{2}$ where $u=\sum_{i=1}^{N_{g}} \sum_{j=1}^{N_{s}} P(i,j)i$  Zone Variance $\sum_{i=1}^{N_{g}} \sum_{j=1}^{N_{s}} P(i,j){(j-u)}^{2}$ where $u=\sum_{i=1}^{N_{g}} \sum_{j=1}^{N_{s}} P(i,j)j$  Gray Level Non-uniformity Normalized $\frac{\sum_{i=1}^{N_{g}} {(\sum_{j=1}^{N_{s}} P(i,j))}^{2}}{\sum_{i=1}^{N_{g}} \sum_{j=1}^{N_{s}} {P(i,j)}^{2}}$  Size Zone Non-uniformity Normalized $\frac{\sum_{j=1}^{N_{s}} {(\sum_{i=1}^{N_{g}} P(i,j))}^{2}}{\sum_{i=1}^{N_{g}} \sum_{j=1}^{N_{s}} {P(i,j)}^{2}}$  Zone Entropy $-\sum_{i=1}^{N_{g}} \sum_{j=1}^{N_{s}} P(i,j)\log_{2} (P(i,j)+\epsilon)$  where $P(i,j)$ is the size zone matrix, $N_{g}$ is the number of intensity levels, $N_{s}$ is the number of zone sizes, $N_{p}$ is the number of voxels, and $\epsilon$ is an arbitrary small positive number. |
